# Evaluation of Unconventional Protein Secretion by *Saccharomyces cerevisiae* and other Fungi

**DOI:** 10.3390/cells7090128

**Published:** 2018-08-31

**Authors:** Natsuko Miura, Mitsuyoshi Ueda

**Affiliations:** 1 Graduate School of Life and Environmental Sciences, Osaka Prefecture University, Sakai 599-8531, Japan; 2 Graduate School of Agriculture, Kyoto University, Kyoto 606-8502, Japan; miueda@kais.kyoto-u.ac.jp

**Keywords:** protein secretion, *Saccharomyces cerevisiae*, unconventional secretion pathway, fungal allergens

## Abstract

Development of proteome analysis of extracellular proteins has revealed that a wide variety of proteins, including fungal allergens are present outside the cell. These secreted allergens often do not contain known secretion signal sequences. Recent research progress shows that some fungal allergens are secreted by unconventional secretion pathways, including autophagy- and extracellular-vesicle-dependent pathways. However, secretion pathways remain unknown for the majority of extracellular proteins. This review summarizes recent data on unconventional protein secretion in *Saccharomyces cerevisiae* and other fungi. Particularly, methods for evaluating unconventional protein secretion are proposed for fungal species, including *S. cerevisiae*, a popular model organism for investigating protein secretion pathways.

## 1. Introduction

Protein secretion is an essential way for cells to communicate with extracellular environments and/or other cells. In fungi, proteins transported to extracellular space sometimes work as allergens [1,2,3] or virulent factors [4,5]. Previously reported fungal allergens include a number of secreted proteins [1]. Some fungal protein allergens do not contain known secretory signal peptides (SPs) as used in the conventional protein secretion (CPS) pathway [6,7]. These proteins without SPs have been regarded as intracellular allergens [1]. Recent advances in secretome analysis have revealed that metabolic enzymes and heat shock proteins (HSPs) are secreted without known SPs, suggesting important roles of non-CPS pathways in fungal pathogenesis [8,9,10,11,12,13,14]. In this review, first, we provide an overview on conventional and unconventional protein secretion (UPS) machineries, as well as on proteins secreted via UPS studied in several fungi, including *Saccharomyces cerevisiae*. Detailed molecular machineries of CPS and UPS can be found elsewhere in several excellent reviews [15,16,17]. Next, we discuss approaches to evaluate UPS in fungi. In addition, we present an overview on currently available proteome databases of unconventionally secreted proteins, and use of the databases. Finally, we summarize unconventionally secreted fungal allergens based on previous secretome data.

## 2. Overview on Conventional and Unconventional Protein Secretions in Fungal Cells

While the CPS machinery is well documented in fungi, the UPS machinery is less well known since there might be several UPS pathways, possibly independent of each other. For some proteins, including Acyl-CoA-binding protein Acb1p, superoxide dismutase Sod1p, and the glycolytic enzyme enolase, novel unconventional secretion pathways are studied. There are also other unconventionally secreted proteins, including which are validated to be secreted. Further research is awaited to reveal the machineries of UPS.

### 2.1. Conventional Protein Secretion in Fungal Cells

The CPS pathway is a vesicle-mediated protein transport pathway through which proteins are translocated from the endoplasmic reticulum (ER) and Golgi towards secretion vesicles before their extra-cellular secretion. After or in the course of translation, proteins are translocated to protein transporter complexes on the ER membrane [15]. 

The presence of an N-terminal SP is the most significant difference between conventionally and unconventionally secreted proteins. The N-terminal sequence is used for transporting the protein to the ER membrane and is cleaved after translocation into the ER [15]. Using SignalP [18,19], PSort [20,21], TargetP [22,23], or relevant software, the secretion signal sequence is predictable and it is easier to predict conventionally secreted proteins based on the fungal genome or using transcriptome data [24]. 

### 2.2. Machineries of Unconventional Protein Secretion in Fungal Cells

Proteins secreted via the UPS pathway are extracellular proteins that do not contain known SPs. Vesicle-mediated secretion is the most investigated pathway for UPS in fungal cells [25]. Given that most of the major pathogenic fungal genomes are sequenced [26] and methods for isolating intercellular vesicles are available [27,28], molecular machineries for UPS have been proposed in several fungal species. For example, cytoplasmic proteins, including HSP70 and enolase were detected in extracellular vesicles isolated from *Candida albicans* [11], *Cryptococcus neoformans* [29], and *Paracoccidioides brasiliensis* [30], as well as in *S. cerevisiae* [31]. As the roles of extracellular vesicles in fungal infection were recognized [32,33,34], some studies employed comparative analysis of the secretome of pathogenic fungi and *S. cerevisiae* [9,30]. Vallejo and colleagues identified 72 *P. brasiliensis* proteins from extracellular vesicles commonly found in at least two other fungal species [30]. Among these 72 *P. brasiliensis* proteins, 67 were identified previously in *S. cerevisiae* extracellular vesicles [31], suggesting common secretion machineries among fungal species.

**Figure 1:**
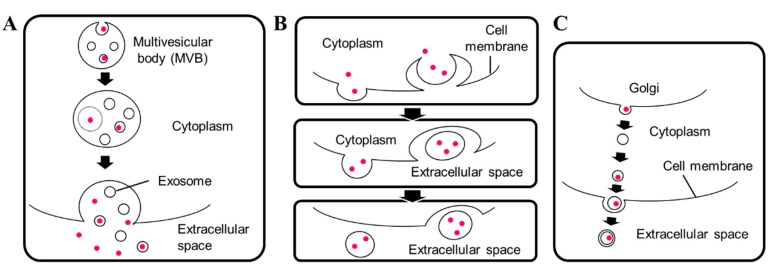
Different types of extracellular vesicle secretion in fungi. Prepared in reference with previous reports [25,52]. (**A**) Exosomes secreted via fusion of multivesicular body with plasma membranes; (**B**) microvesicles budding from cytoplasm; (**C**) Golgi-involved production of membrane vesicles. Pink dot: unconventionally secreted proteins.

Bilayered extracellular vesicles in fungal species were characterized previously [9]. In *S. cerevisiae*, the diameters of extracellular vesicles range from 50 to 250 nm [31]. In 2015, Kabani and Melki reported vesicles with diameters ranging from 30 to 100 nm containing the fungal prion protein Sup35p [35]. Meanwhile, several types of extracellular vesicles have been described [25,36]. They include exosomes secreted via fusion of the multivesicular body with the plasma membrane [31], microvesicles budding from cytoplasm [37], and Golgi-involved production of membrane vesicles [31], in which vesicles may be transferred through other membrane structures such as early or late endosomes, as seen in Figure 1. 

Whether a protein is secreted via a single pathway and how the secreted vesicles pass through the fungal cell wall remains unknown [25,38]. It should be noted that some extracellular vesicle-dependently secreted proteins, including HSP70 and enolase, were also found in non-vesicular fractions in *P. brasiliensis* [30], suggesting that single proteins are secreted via multiple unconventional secretion pathways. While the mechanisms for sorting proteins and directing them to different vesicles are unknown, environmental stimuli is suggested to induce changes in components of extracellular vesicles [39]. It has been reported that environmental stimuli such as glucose concentration induce changes in *S. cerevisiae* extracellular vesicles [40], in particular, glucose starvation induces secretion of extracellular vesicles [41]. It has been reported that extracellular vesicles of *C. neoformans* stay in the cell wall [42]. Alternatively, it may be possible to block the secretion of specific extracellular vesicles by targeting molecules specific to the target vesicle, as suggested by Matos Baltazar and colleagues [43]. Taken together, extracellular vesicles are not uniform in their size, nor in their components and final destination.

#### 2.2.1. Unconventional Secretion of Acb1 and Sod1p in Fungal Cells 

Although unconventional secretory machineries are mostly unknown, the UPS pathways of several unconventionally secreted proteins are relatively well investigated. For example, the secretion of Acb1p is autophagosome-dependent [44] via an autophagosome-mediated cellular component [45] called a compartment for unconventional protein secretion (CUPS) [46]. Cytoplasmic Acb1p is recruited into CUPS by Grh1p, then compartmentalized into vesicles, and transported by endosomes. The endosome compartments are incorporated into a multivesicular body (MVB) by an endosomal sorting complex required for transport (ESCRT)-dependent processes. Then, MVB is fused with soluble *N*-ethylmaleimide-sensitive factor attachment protein receptor (SNARE) proteins such as Sso1p [17]. 

The secreted Acb1 contributes to morphological changes in *C. neoformans* [47]. Recently, Sod1p was found to be secreted via the same machinery as Acb1p [48]. Interestingly, both Acb1p and Sod1p have Asp-Glu di-acidic motifs and substitution of these residues to alanine partially inhibits their secretion [48]. These findings suggest that there are protein domains or motifs that specifically promote unconventional secretion, many of which remain to be identified. 

#### 2.2.2. Unconventional Secretion of Glycolytic Enzymes 

The glycolytic enzyme enolase is a protein for which the secretion pathway remains to be clarified. While enolase has been found in extracellular vesicles of *C. albicans* [11] and *S. cerevisiae* [31], the secretion of *S. cerevisiae* enolase, or Eno2p, is a SNARE protein Tlg2p-dependent, while it is autophagy-related protein- independent [14]. Moreover, several amino acid sequences or domains that are related to the unconventional secretion of enolase have been reported [14,49,50,51]. Some unconventionally secreted proteins are possibly secreted via more than one pathway [30], and this might be the case for enolase. It would be important to develop markers and/or mutant cell lines in which specific secretion pathways can be investigated. 

## 3. Approaches for Evaluating Unconventional Protein Secretion 

Fungal proteomics have identified various unconventionally secreted proteins, including some related to fungal allergens. While the presence of metabolic enzymes in the extracellular space can be caused by cell death or leakage, the presence of enolase among secreted proteins has been gradually accepted as not accidental [53].

Recently, Vivek-Ananth and colleagues performed *in silico* analysis on proteome datasets of opportunistic fungal pathogens, including *Aspergillus fumigatus* and ten other *Aspergillus* species. They developed a workflow to determine whether the proteins are secreted via conventional or unconventional secretion pathways. They estimated that in *A. fumigatus*, approximately 0.65% of the proteome is secreted via unconventional secretion and 6.1% of the proteome is secreted via conventional secretion, represented with 64 unconventionally secreted proteins and 598 conventionally secreted proteins, respectively [54]. 

### 3.1. Methods of Secretome Analysis

Several steps are required for identifying and evaluating UPS in fungal cells. Although fungi usually have a thick cell wall, intracellular proteins easily leak because of mechanical stress and cell death. In *S. cerevisiae*, the total number of protein molecules per cell is ~5.0 × 10^7^ [55], while the number of Eno2p molecules per cell is ~9.3 × 10^5^ [56], representing up to 2% of total proteins. Among other proteins, the numbers of actin (Act1p), Acb1p, Sod1p, and heat shock protein 70 (SSA1p) molecules per cell are ~1.2 × 10^5^, ~1.4 × 10^5^, ~1.2 × 10^5^, and ~3.7 × 10^5^, respectively [56]. Abundant proteins in the cell are detected easily in extracellular space when cell membranes are disturbed. Therefore, it is essential to construct methods for preparing samples carefully. Some validated unconventionally secreted proteins may be used as positive controls for unconventional secretion. On the other hand, green fluorescent proteins (GFP) and their derivatives are not secreted and could be used as negative controls [14,57]. It is essential to use these proteins for quality control when samples are prepared for tests [52]. The method below could be implemented to evaluate UPS:

1. Prepare extracellular protein samples
2. Test the samples by SDS-PAGE and Western blotting
3. Non-targeted proteome analysis of secreted proteins
4. Reconstruction of the UPS on specific proteins for further analysis

In the first step, extracellular proteins are prepared according to the purpose of research. Detailed methods for sample preparation can be found in previous reports [28,52]. Samples should be carefully prepared, avoiding unnecessary physical or chemical stresses to the cells. It is highly recommended, especially when the experiment is done for the first time, to use a proven intracellular protein such as GFP, as a standard to evaluate leakage. 

In the second step, prepared proteins are tested by SDS-PAGE and Western blotting to validate contamination of leaked proteins into the samples. Antibodies against validated unconventionally secreted proteins, as well as negative controls, such as GFP, can be used.

In the third step, proteins are processed and detected by 2-DE followed by MS analysis, LC-MS/MS analysis, or other relevant methods. Since a number of proteome data are available, the beginning of this step can be substituted by *in silico* analysis of previous results, provided the methods used for protein preparation are carefully checked for each case. It also should be noted that it does not mean deposited proteins are secreted at any time. It has been reported that nutrient starvation changes the secretome of fungi [46]. In addition, proteomic approaches are based on the number of detectable proteins or peptide fragments, meaning that proteins with low abundance are likely not detected. 

In the final step, unconventional secretion of specific proteins should be validated. Reconstruction of unconventional secretion using plasmids [52] would be effective to adjust protein levels in the cells, to validate secretion, and to determine the detailed secretion pathway of the target proteins.

### 3.2. Proteome Databases

Secretome data are usually deposited to proteome databases such as ProteomeXchange [58], which is a consortium of global proteome databases [59,60], including PRIDE [61,62], PeptideAtlas [63,64], MassIVE [65], jPOST [66,67], iProX [68], and Panorama [69,70]. Because of standardization of data formats and experimental workflows, the deposited data are becoming increasingly accessible and reusable [71]. Fungal secreted proteins can be found easily by species-specific searches of the database. Currently, since secreted proteins of pathogenic and allergenic fungi are often harmful to host cells, the importance of fungal secretome data is widely accepted [72]. This awareness possibly accelerates the deposition and utilization of proteome databases.

### 3.3. Re-Analysis of Fungal Secretome

There are several ways of re-analyzing previous secretome data, including manual downloading and sorting of excel files submitted along with published reports. Since there are less reports for which the proteome data are submitted to proteome databases than there are with data attached as excel files or tables as shown in Table 1, manual treatment of the data as well as development of custom software is still effective. For the data available through the PRIDE database, due to recent updates, re-analysis of the data is becoming easier [73]. 

For re-analyzing proteome data deposited to PRIDE database, PRIDE Inspector [74,75] is used. After installation, PRIDE Inspector can be used on personal devices. By downloading the data after searching with ID as seen in Table 1, the data can be visualized on the software. However, it is still difficult to comparatively analyze results from different projects, because experimental conditions, methods for preparation, databases used for analysis, and instrumental settings vary dependent on each project. Still, since raw data is submitted to the database, re-analyzing several data on the same platform is becoming easier. 

Table 2 shows the commonly detected extracellular proteins in four previous reports [14,31,40,76]. In addition to Acb1p and Sod1p discussed previously, the proteins listed are possibly unconventionally secreted proteins.

## 4. Unconventionally Secreted Fungal Allergens 

Most fungal allergens are extracellular proteins. Among previously reported fungal allergens, 18 do not contain known SPs used in the CPS pathway [6,7], shown in Table 3. Initially, these proteins without SPs were considered intracellular allergens [1]. Proteomic analyses detected these proteins in several fungal species as seen in Table 4, and they are possibly unconventionally secreted. Among fungal allergens, superoxide dismutase (SOD) and enolase are the only unconventionally secreted proteins for which the secretion machineries are studied. 

## 5. Discussion and Future Perspectives

Fungal extracellular vesicles as vectors for unconventionally secreted proteins attract increased attention because of their potential roles in host-microbe interactions [32,34,39]. Since secretome analysis in addition to genome editing of fungal cells are becoming popular, efforts to regulate fungal secretome [128] would be accelerated. In terms of fungal extracellular proteins as allergens, the importance of extracellular vesicles as cell wall components should be noted [39], since vesicles on the cell wall allow exposure of host cells with concentrated proteins when the vesicle is ruptured. 

Notably, most of the unconventionally secreted proteins related to fungal allergens are also reported in *S. cerevisiae* cells, as seen in Table 4. *S. cerevisiae* is a model organism extensively studied for understanding the conventional secretion pathway, and mutants in regulatory molecules important for conventional secretion are easily available [129,130,131,132]. With genome-wide knockout [133,134], overexpression [135], and knockdown [135,136] libraries, *S. cerevisiae* remains an attractive model for investigating unconventional secretion pathway of proteins, including fungal allergens [14,52,137].

The use of extracellular vesicles for therapeutics [138] is a possible way of utilizing fungal extracellular vesicles. For the purpose, it would be important to increase the number of extracellular vesicles secreted by fungi, as unconventional secretion is not a major secretory pathway in fungal cells [54].

Fungal unconventional secretion has been driven by the development of proteome analysis [10,11]. Consequent development of the database and related tools [59] now assists the comparative systems analysis of numerous secretome data as reported recently [54]. Accumulating further data would enable classification of unconventional secretion pathways, with the development of mutant strains in which a specific pathway is deficient. These developments would clarify species-dependent unconventional secretion pathways, providing insights into strategies of pathogenic fungi in the future. 

## Figures and Tables

**Table 1:** Recent proteome data of extracellular proteins in some allergenic and/or pathogenic fungi.

Major Fungi with Known Allergens	Number of Extracellular Proteins Identified	Method ^1^	Ref.	Year	Database ^2^	ID
*Alternaria alternata*	1383	LC-MS/MS	[77]	2016	-	-
	1315	iTRAQ	[78]	2017	-	-
	95	2-DE	[79]	2018	-	-
*Aspergillus fumigatus*	64	2-DE	[80]	2011	-	-
	128	SDS-PAGE, LC-MS/MS	[81]	2018	-	-
	437	In sillico analysis of previous secretome data	[54]	2018	-	-
*Beauveria bassiana*	13	2-DE	[82]	2007	-	-
	50	LC-MS/MS	[83]	2016	-	-
*Candida albicans*	27	2-DE	[84]	2002	W2	-
	14	LC-MS/MS	[85]	2004	-	-
	48	2-DE	[86]	2006	-	-
	50	LC-MS/MS	[87]	2009	-	-
	143	LC-MS/MS	[88]	2010	-	-
	84	LC-MS/MS	[89]	2011	-	-
	41	LC-MS/MS	[90]	2012	PX	PXD000008
	96	LC-MS/MS	[11]	2015	PX	PXD000525
	170	LC-MS/MS	[91]	2015	PX	PXD000525
*Saccharomyces cerevisiae*	99	2-DE LC-MS/MS	[92]	2010	-	-
	219	LC-MS/MS	[93]	2010	-	-
	127	LC-MS/MS	[77]	2010	-	-
	42	LC-MS/MS	[78]	2011	-	-
	42	LC-MS	[79]	2012	P	PRD000729
	347	iTRAQ	[80]	2014	-	-
	694	2-DE MALDI-TOF/TOF LC-MS/MS	[81]	2015	PX	PXD001133

^1^ 2-DE: two-dimensional gel electrophoresis, iTRAQ: quantitative proteomics using isotope-coded protein labels. ^2^ PX: Proteome Xchange [58], P: PRIDE [61], W2: WORLD-2DPAGE List [94], -: not specified.

**Table 2:** Extracellular proteins of *S. cerevisiae* commonly detected by four independent analysis [14,31,40,76].

Cellular Process	Description	Accession Number
Carbohydrate Metabolism	Cdc19p, Pyruvate kinase	gi|6319279
	Eno1p, Enolase	gi|6321693
	Eno2p, Enolase II	gi|6321968
	Pdc1p, Major pyruvate decarboxylase	gi|6323073
	Pgk1p, 3-Phosphoglycerate kinase	gi|10383781
	Tdh3p, Glyceraldehyde-3-phosphate dehydrogenase	gi|6321631
Protein Folding	Ssa1p, Hsp70 family	gi|144228166
Other Functions	Tif2p, Translation initiation factor eIF4A	gi|6322323

**Table 3:** Fungal allergens without known secretion signal sequences.

Classification	Allergens	Species	Genbank Accession No.	Ref.
Translation	Acid ribosomal protein P1	*Alternaria alternata*	X84216	[95]
		*Cladosporium herbarum*	X85180	[96]
		*Penicillium brevicompactum*	AY786077	[97]
	Acid ribosomal protein P2	*Alternaria alternata*	X78222, U87806	[95]
		*Aspergillus fumigatus*	AJ224333	[98]
		*Cladosporium herbarum*	X78223	[95,99]
		*Fusarium culmorum*	AY077706	[100]
	L3 ribosomal protein	*Aspergillus fumigatus*	AF464911	[101]
	Elongation factor 1 beta	*Penicillium citrinum*	AY363911	[102]
Metabolism	Alcohol dehydrogenase	*Candida albicans*	X81694	[103]
	Aldehyde dehydrogenase	*Alternaria alternata*	X78227, P42041	[95]
		*Beauveria bassiana*	DQ767721	[104]
		*Cladosporium herbarum*	X78228	[95]
	Enolase	*Alternaria alternata*	U82437	[105]
		*Aspergillus fumigatus*	AF284645	[106]
		*Beauveria bassiana*	DQ767719	[104]
		*Candida albicans*	L04943	[107,108]
		*Cladosporium herbarum*	X78226	[95]
		*Curvularia lunata*	AY034826	[109]
		*Penicillium citrinum*	AF254643	[106]
		*Saccharomyces cerevisiae*	J01322	[106,110]
		*Rhodotorula mucilaginosa*	AY547285	[111]
	Formate dehydrogenase	*Candida boidinii*	AJ011046	[112]
	Mannitol dehydrogenase	*Alternaria alternata*	AY191815	[113]
		*Cladosporium herbarum*	AY191816	[114]
	Mitochondrial malate dehydrogenase	*Malassezia furfur*	AF084828	[115]
Heat shock proteins	HSP70	*Alternaria alternata*	U87807, U87808	[116]
		*Cladosporium herbarum*	X81860	[117]
		*Penicillium citrinum*	U64207	[118]
	HSP88	*Malassezia sympodialis*	AJ428052	[119,120]
	HSP90	*Aspergillus fumigatus*	U92465	[121]
Others	MnSOD	*Aspergillus fumigatus*	U53561	[122]
		*Saccharomyces cerevisiae*	X02156	[123]
		*Malassezia sympodialis*	AJ548421	[120]
	Peptidyl-prolyl isomerase	*Aspergillus fumigatus*	AJ006689	[124]
	Protein disulfide isomerase	*Alternaria alternata*	X84217	[95]
	Thioredoxin-like protein	*Fusarium culmorum*	AY077707	[100]
	GST	*Alternaria alternata*	AY514673	[125]

Prepared with reference to previous reports [1,126]. Absence of secretion signal peptide was determined by SignalP 4.1 [127]. HSP: heat shock proteins, SOD: superoxide dismutase, GST: Glutathione S-transferase.

**Table 4:** Fungal allergen-related proteins detected by secretome analysis.

Classification	Secreted Protein	Species	Ref.
Metabolism	Alcohol dehydrogenase	*Alternaria alternata*	[78]
		*Candida albicans*	[84,91]
		*Saccharomyces cerevisiae*	[31,40,76]
	Aldehyde dehydrogenase	*Candida albicans*	[91]
		*Saccharomyces cerevisiae*	[31,40]
	Enolase	*Aspergillus fumigatus*	[80]
		*Candida albicans*	[11,84,89,91]
		*Saccharomyces cerevisiae*	[14,31,40,76,93]
	Formate dehydrogenase	*Aspergillus fumigatus*	[80]
		*Candida albicans*	[91]
	Mitochondrial malate dehydrogenase	*Aspergillus fumigatus*	[80]
		*Saccharomyces cerevisiae*	[14,40]
Heat shock proteins	HSP70	*Candida albicans*	[11,91]
		*Saccharomyces cerevisiae*	[31,40]
	HSP90	*Candida albicans*	[84,89]
		*Saccharomyces cerevisiae*	[14,31,40]
Others	SOD	*Aspergillus fumigatus*	[80]
		*Saccharomyces cerevisiae*	[14,31,40,76]
	Peptidyl-prolyl isomerase	*Candida albicans*	[91]
		*Saccharomyces cerevisiae*	[14,31,40]
	Protein disulfide isomerase	*Candida albicans*	[88]
		*Saccharomyces cerevisiae*	[14,31]
	Thioredoxin	*Candida albicans*	[91]
		*Saccharomyces cerevisiae*	[14,31,40]
	GST	*Saccharomyces cerevisiae*	[31]

HSP: heat shock proteins, SOD: superoxide dismutase, GST: Glutathione S-transferase.
